# EGFR mutations are associated with favorable intracranial response and progression-free survival following brain irradiation in non-small cell lung cancer patients with brain metastases

**DOI:** 10.1186/1748-717X-7-181

**Published:** 2012-10-30

**Authors:** Hsin-Lun Lee, Tao-Sang Chung, Lai-Lei Ting, Jo-Ting Tsai, Shang-Wen Chen, Jeng-Fong Chiou, Henry Wing-Cheung Leung, H Eugene Liu

**Affiliations:** 1 Department of Radiation Oncology, Wan Fang Hospital, Taipei Medical University, Taipei, Taiwan; 2 Department of Internal Medicine, Wan Fang Hospital, Taipei Medical University, Taipei, Taiwan; 3 Department of Radiation Oncology, Landseed Hospital, Pingzhen, Taiwan; 4 Department of Radiation Oncology, Taipei Medical University Hospital, Taichung, Taiwan; 5 Department of Radiation Oncology, Taipei Medical University-Shuang Ho Hospital, Taipei, Taiwan; 6 Department of Radiation Oncology, China Medical University Hospital, Taichung, Taiwan; 7 Graduate Institute of Clinical Medicine, Taipei Medical University, 250 Wushing Street, Taipei, Taiwan

**Keywords:** Epidermal growth factor receptor, Non-small cell lung cancer, Brain metastases, Radiotherapy

## Abstract

**Background:**

The presence of epidermal growth factor receptor (EGFR) mutations in non-small cell lung cancer (NSCLC) is associated with increased radiosensitivity *in vitro*. However, the results from clinical studies regarding the radiosensitivity in NSCLC with mutant EGFR are inconclusive. We retrospectively analyzed our NSCLC patients who had been regularly followed up by imaging studies after irradiation for brain metastases, and investigated the impact of EGFR mutations on radiotherapy (RT).

**Methods:**

Forty-three patients with brain metastases treated with RT, together with EGFR mutation status, demographics, smoking history, performance status, recursive partitioning analysis (RPA) class, tumor characteristics, and treatment modalities, were included. Radiological images were taken at 1 to 3 months after RT, and 3 to 6 months thereafter. Radiographic response was evaluated by RECIST criteria version 1.1 according to the intracranial images before and after RT. Log-rank test and Cox regression model were used to correlate EGFR mutation status and other clinical features with intracranial radiological progression-free survival (RPFS) and overall survival (OS).

**Results:**

The median follow-up duration was 15 months. Patients with mutant EGFR had higher response rates to brain RT than those with wild-type EGFR (80% vs. 46%; *p* = 0.037). Logistic regression analysis showed that EGFR mutation status is the only predictor for treatment response (*p* = 0.032). The median intracranial RPFS was 18 months (95% CI = 8.33-27.68 months). In Cox regression analysis, mutant EGFR (*p* = 0.025) and lower RPA class (*p* = 0.026) were associated with longer intracranial RPFS. EGFR mutation status (*p* = 0.061) and performance status (*p* = 0.076) had a trend to predict OS.

**Conclusions:**

Mutant EGFR in NSCLC patients is an independent prognostic factor for better treatment response and longer intracranial RPFS following RT for brain metastases.

## Background

Non-small cell lung cancer (NSCLC) is the leading cause of mortality from cancer worldwide and the most common cancer responsible for brain metastases [1,2]. In NSCLC patients with brain metastases, radiotherapy (RT), the cornerstone of treatment, has yielded response rates of 50% to 75% for intracranial lesions [3]. Many prognostic factors have been explored in patients with brain metastases [4-12] including age, performance status, control of primary tumor, extent of extracranial disease, primary site of cancer, number of brain metastases, and treatment modalities. These parameters have been incorporated in commonly used indices in radiation oncology such as the Radiation Therapy Oncology Group (RTOG) recursive partitioning analysis (RPA) class and the Graded Prognostic Assessment (GPA). In addition, incorporation of biomarkers such as the expression level of epidermal growth factor receptor (EGFR), vascular endothelial growth factor, and cyclooxygenase-2 has also been correlated with treatment outcome after RT [13]. Among these biomarkers, amplification of EGFR has been extensively studied and is regarded as a poor prognostic factor in cancer [13-16]. In contrast to the radioresistance conferred by EGFR overexpression, the radiosensitivity of lung cancer cells with mutant EGFR has been demonstrated *in vitro*[17]. NSCLC cell lines with EGFR mutations are more sensitive to radiation, evidenced by increased apoptosis, than those with wild-type EGFR. However, prior clinical studies [18,19] attempting to investigate the relationship between EGFR mutations and radiosensitivity in NSCLC patients with brain metastases did not reach unanimous conclusions, mostly due to the lack of coordinated follow-up. Therefore, detailed analysis on the response of brain metastases to RT is imperative to clarify the role of EGFR mutations in NSCLC. We retrospectively analyzed our NSCLC patients who had been regularly followed up by imaging studies after irradiation for brain metastases, and investigated the impact of EGFR mutations on RT.

## Methods

### Patient eligibility

We reviewed 246 NSCLC patients who underwent EGFR mutation testing and received cancer treatment at Wan Fang Hospital and Taipei Medical University Hospital between April 2003 and January 2011. Of them, 134 patients were identified to have brain metastases. The presence of EGFR mutations was detected by either direct sequencing or the methods described previously [20]. This study proposal was approved by our institutional review board for the use of the patients’ pathology, medical records, and radiological images. The inclusion criteria for the analysis were as follows: (1) conventional brain RT as the only intracranial treatment; and (2) consecutive brain imaging follow-up 1 to 3 months after RT, and 3 to 6 months thereafter. Either contrast-enhanced brain magnetic resonance imaging (MRI) or computed tomography (CT) was required for assessment of intracranial tumors. A total of 43 eligible patients were enrolled into this study. The histology for most patients (*n* = 40) was adenocarcinoma, whereas 1 patient had adenosquamous carcinoma and 2 patients had poorly differentiated carcinoma.

Clinical characteristics such as age, gender, smoking history, performance status according to the Eastern Cooperative Oncology Group (ECOG), RPA class, extent of disease and the duration of EGFR tyrosine kinase inhibitor (TKI) therapy of each patient were collected by reviewing their medical records. Patients were stratified into RPA prognostic class (I, II, or III) based on the RTOG classification, which consists of age, performance status, control of primary tumor, and presence of extracranial metastases [6]. Controlled primary tumor was defined as no evidence of extracranial disease progression within 1 month before brain RT. Tumor characteristics, including number, size, and presence of hemorrhage, were evaluated on the basis of the pre-treatment intracranial radiological images. Cause of death was determined by the symptoms at last follow-up and/or radiological images within 3 months of death.

### RT and assessment of RT response

The standard treatment used for brain irradiation in this study was whole brain RT with 30 to 40 Gy in 10–20 fractions. Seventeen patients (40%) had local boost to tumor sites up to 50–60 Gy. The radiographic response of intracranial tumors was assessed using the Response Evaluation Criteria in Solid Tumors (RECIST) guideline version 1.1 [21] by comparing the pre- and post-treatment intracranial images. Any in-field tumor progression or the appearance of new malignant lesions denoted progressive disease. A responder was defined as a combination of complete and partial response. Treatment-associated toxicities were scored according to the Common Terminology Criteria for Adverse Events version 3.0 [22].

### Statistical analysis

Categorical data are presented as number (percentage), and continuous data are reported as mean ± standard deviation. Comparison of categorical variables between the mutant and wild-type EGFR groups was carried out by Fisher’s exact test and comparison of continuous variables was performed by independent sample *t*-test. Multivariable logistic regression analysis was used to examine the impact of variables on response rate.

The intracranial radiological progression-free survival (RPFS) was counted from the first day of brain RT to the date of radiological progression or the last radiological documentation of the intracranial disease status. The overall survival (OS) was measured from the first day of brain RT to the date of death or last follow-up. We used the Kaplan–Meier method to calculate the RPFS and the OS. The log-rank test and Cox regression analysis were performed to explore the impact of variables on survival rate. The scheme of multivariable regression models (both logistic and Cox models) was as follows: a series of univariate (unadjusted) regression analyses were performed, and those variables whose *p* value is less than 0.1 in the univariate analyses were then included in the multivariable stepwise logistic regression analyses. Statistical significance was defined as a two-sided *p* value of <0.05. All analyses were carried out using SPSS statistical software (SPSS 15.0; SPSS Inc., Chicago, IL, USA).

## Results

### Patient and tumor characteristics

Of the 43 patients, 30 (70%) had EGFR mutations (15 had exon 19 deletions and 15 had exon 21 L858R point mutation). The patient demographics and tumor characteristics together with EGFR mutation status are listed in Table 1. Consistent with prior studies [23-25], the proportion of females and never-smokers was higher in patients with mutant EGFR (57% and 73%, respectively). The size of the largest lesion was significantly larger in patients with mutant EGFR (24.51 ± 11.74 mm) than those with the wild-type (16.45 ± 5.89 mm) (*p* = 0.024). Hemorrhagic brain metastases (*n* = 11) were observed only in patients with EGFR mutations. Of all patients, nineteen (44%) received EGFR TKI (14 received erlotinib and 5 received gefitinib) during the period of brain RT. The median duration of EGFR TKI therapy in patients with mutant EGFR (*n* = 15) was 215 days (range, 25–412 days), whereas that in patients with the wild-type (*n* = 4) was 32.5 days (range, 11–104 days). Due to limited numbers in the wild-type group, statistical analysis was not performed.

**Table 1:** Clinical characteristics of non-small cell lung cancer patients with brain metastases treated with brain RT stratified by EGFR mutation status

**Characteristics**	**Total(*N *= 43)**	**EGFR mutation status**		***P***
		**Positive(*n *= 30)**	**Negative(*n *= 13)**	
Age, years				0.332
Median (range)	59 (35-83)			
<60	22 (51)	17 (57)	5 (38)	
≧60	21 (49)	13 (43)	8 (62)	
Gender				0.054
Female	20 (47)	17 (57)	3 (23)	
Male	23 (53)	13 (43)	10 (77)	
Smoking history				0.043
Never	27 (63)	22 (73)	5 (38)	
Ever	16 (37)	8 (27)	8 (62)	
ECOG performance status	1.60±0.88	1.60±0.89	1.62±0.87	0.959
RPA class				1.000
Class I	5 (12)	4 (13)	1 (8)	
Class II	31 (72)	21 (70)	10 (77)	
Class III	7 (16)	5 (17)	2 (15)	
Primary tumor status				1.000
Controlled	29 (67)	20 (67)	9 (69)	
Uncontrolled	14 (33)	10 (33)	4 (31)	
Extracranial metastases				1.000
Absent	12 (28)	8 (27)	4 (31)	
Present	31 (72)	22 (73)	9 (69)	
Number of BM				0.491
≦3	14 (33)	11 (37)	3 (23)	
>3	29 (67)	19 (63)	10 (77)	
Size of largest BM (mm)	22.07±10.91	24.51±11.74	16.45±5.89	0.024
Hemorrhagic BM				0.019
No	32 (74)	19 (63)	13 (100)	
Yes	11 (26)	11 (37)	0 (0)	
Total dose				0.310
≦40 Gy_2_	26 (60)	20 (67)	6 (46)	
>40 Gy_2_	17 (40)	10 (33)	7 (54)	
EGFR TKI during RT				0.324
No	24 (56)	15 (50)	9 (69)	
Yes	19 (44)	15 (50)	4 (31)	
Type of EGFR TKI				1.000
Gefitinib	5 (26)	4 (27)	1 (25)	
Erlotinib	14 (74)	11 (73)	3 (75)	
Chemotherapy during RT				1.000
No	33 (77)	23 (77)	10 (77)	
Yes	10 (23)	7 (23)	3 (23)	

Abbreviations: *ECOG* Eastern Cooperative Oncology Group, *RPA* recursive partitioning analysis, *BM* brain metastases, *Gy*_*2*_ biologically equivalent dose equal to fraction size of 2 Gy, *TKI* tyrosine kinase inhibitor.

Categorical data were presented as number (percentage) and continuous data were presented as mean ± standard deviation.

### Radiographic response to RT

Of the 43 patients, 5 had a complete response and 25 had a partial response to RT. The overall response rate was 70%. The rest of the patients either remained stationary in tumor size (*n* = 11) or had progressive intracranial lesions (*n* = 2). The response rate was significantly higher in patients with EGFR mutations than those with the wild-type (80% vs. 46%; *p* = 0.037, Additional file 1: Table S1). Table 2 shows the association between clinical features and radiographic response to brain RT. Multivariable analyses revealed that EGFR mutation status is the only predictor for treatment response (odds ratio: 4.67, 95% confidence interval [CI]: 1.14–19.12; *p* = 0.032). There was no significant association between radiographic response and treatment modalities such as RT dose and the use of concurrent systemic chemotherapy or EGFR TKI.

**Table 2:** Univariate and multivariable analyses of clinical characteristics in predicting radiographic response to brain RT

**Characteristics**	**Univariate analysis**			**Multivariable analyses**		
	**OR**	**95% CI of OR**	***P***	**OR**	**95% CI of OR**	***P***
Age, years (≧60 vs. <60)	0.48	0.13 to 1.81	0.277			
Gender (male vs. female)	0.98	0.27 to 3.61	0.975			
Smoking history (ever vs. never)	0.93	0.24 to 3.54	0.911			
EGFR mutation status (positive vs. negative)	4.67	1.14 to 19.12	0.032	4.67	1.14 to 19.12	0.032
Mutant EGFR^†^ (exon 21 vs. exon 19)	0.42	0.06 to 2.77	0.369			
ECOG performance status	0.73	0.34 to 1.56	0.415			
RPA class						
I (reference)	1	--	--			
II	0.00	0.00 to ∞	1.000			
III	0.00	0.00 to ∞	1.000			
Primary tumor status (uncontrolled vs. controlled)	1.12	0.28 to 4.57	0.869			
Extracranial metastases (present vs. absent)	2.05	0.51 to 8.34	0.314			
Number of BM (>3 vs. ≦3)	0.89	0.22 to 3.61	0.869			
Size of largest BM (mm)	1.07	0.99 to 1.15	0.091			
Hemorrhagic BM (yes vs. no)	6.00	0.68 to 52.90	0.107			
Total dose (>40 Gy_2_ vs. ≦40 Gy_2_)	1.07	0.28 to 4.05	0.925			
EGFR TKI during RT (yes vs. no)	0.89	0.24 to 3.30	0.864			
Type of EGFR TKI^§^ (erlotinib vs. gefitinib)	1.67	0.20 to 14.05	0.639			
Chemotherapy during RT (yes vs. no)	2.00	0.36 to 11.06	0.427			

Abbreviations: *OR* odds ratio, *CI* confidence interval, *ECOG* Eastern Cooperative Oncology Group, *RPA* recursive partitioning analysis, *BM* brain metastases, *Gy*_*2*_ biologically equivalent dose equal to fraction size of 2 Gy, *TKI* tyrosine kinase inhibitor.

† *n* = 30.

§ *n* = 19.

### Outcome and survival

**Figure 1:**
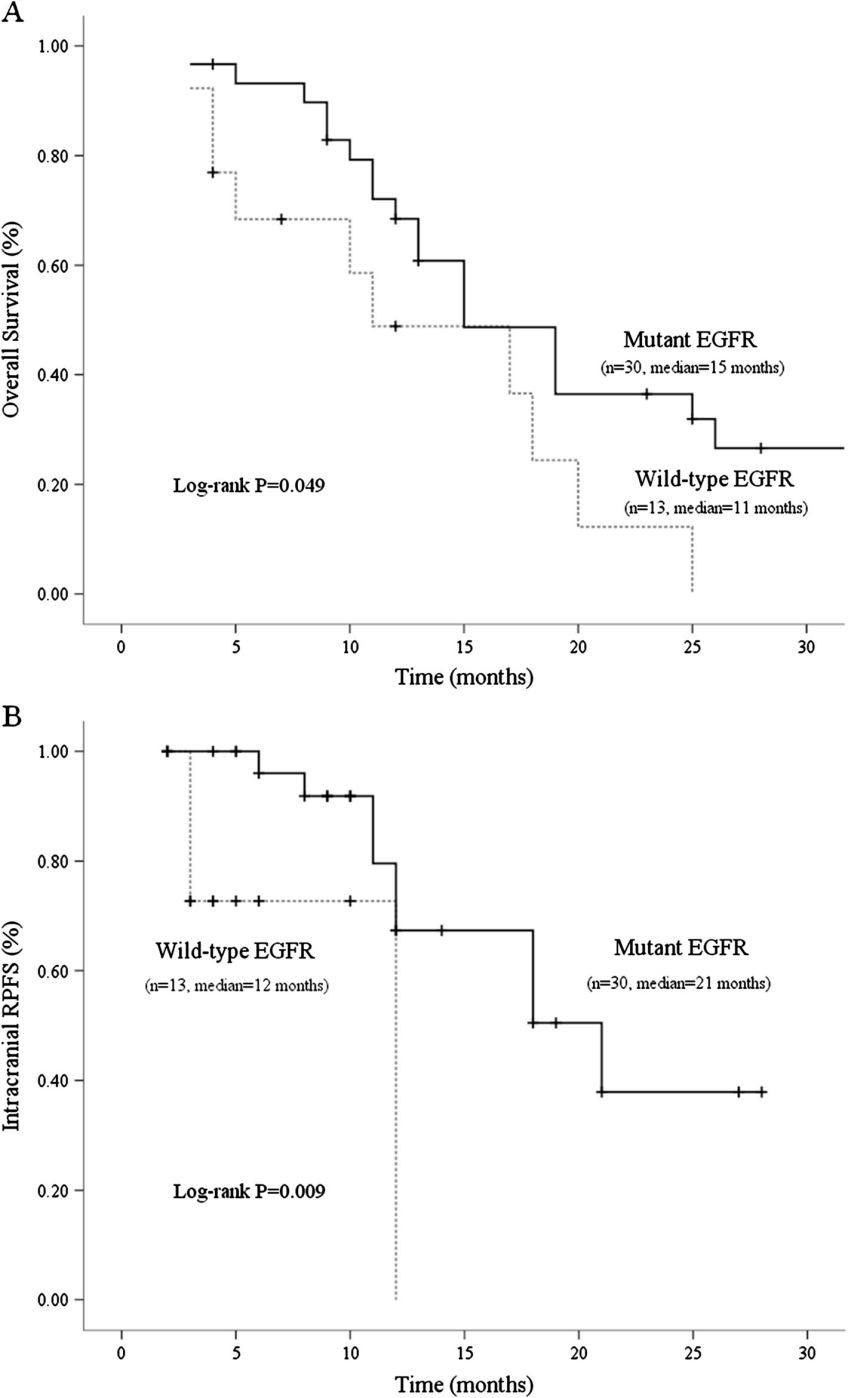
**Survival of non-small cell lung cancer patients with brain metastases treated with brain RT stratified by EGFR mutation status. (A) Overall survival.** (**B**) Intracranial radiological progression-free survival (RPFS). Censored cases is marked by cross sign "+".

There were no ≥grade-3 RT-related toxicities in patients treated with brain RT alone, but 4 patients experienced major toxicities (grade-3 acneiform rash in 2 patients, grade-3 oral mucositis in 1 patient, and grade-3 otitis media in 1 patient) during the course of concurrent EGFR TKI use. To minimize brain edema, oral or intravenous corticosteroids were administered in 39 patients (91%) during the course of brain RT, and tapered off thereafter. After a median follow-up of 15 months (range: 3–39 months), 33 patients had died. Three died of intracranial disease progression, 22 of extracranial disease progression, and 8 of other causes (4 with pneumonia, 1 with urosepsis, 1 with intra-abdominal infection, 1 with upper gastrointestinal bleeding, and 1 with stroke). The median overall survival was 15 months (95% CI: 9.61–20.39 months). The univariate analysis showed that EGFR mutations (*p* = 0.061) and performance status (*p* = 0.076) had a borderline impact in predicting OS (Additional file 2: Table S2]). The median OS for patients with or without EGFR mutations was 15 and 11 months, respectively (Figure 1A). Multivariable analyses did not reveal any other clinical characteristics significantly associated with OS.

Forty-one patients (95%) had at least 2 consecutive follow-up images. Eleven patients (26%) were found to have intracranial recurrence. The median intracranial RPFS was 18 months (95% CI: 8.33–27.68 months). Table 3 summarizes the association between different variables and intracranial RPFS. Multivariable analyses revealed that EGFR mutations (hazard ratio: 0.20, 95% CI: 0.05–0.81; *p* = 0.025) and lower RPA class (*p* = 0.026) are two predictors for longer intracranial RPFS. The median intracranial RPFS was 21 months for patients with EGFR mutations and 12 months for those without (Figure 1B). Also illustrated in Figure 1B, the 1-year RPFS for patients with or without EGFR mutations was 66% and 0% (*p* = 0.009), respectively.

**Table 3:** Univariate and multivariable analyses of clinical characteristics in predicting intracranial radiological progression-free survival

**Characteristics**	**Univariate analysis**			**Multivariable analyses**		
	**HR**	**95% CI of HR**	***P***	**HR**	**95% CI of HR**	***P***
Age, years (≧60 vs. <60)	1.07	0.35 to 3.32	0.904			
Gender (male vs. female)	1.27	0.42 to 3.84	0.673			
Smoking history (ever vs. never)	1.59	0.51 to 4.94	0.419			
EGFR mutation status (positive vs. negative)	0.20	0.05 to 0.77	0.020	0.20	0.05 to 0.81	0.025
Mutant EGFR^†^ (exon 21 vs. exon 19)	1.49	0.40 to 5.61	0.555			
ECOG performance status	1.66	0.84 to 3.27	0.144			
RPA class			0.022^‡^			0.026‡
Class I (reference)	1	--	--	1	--	--
Class II	3.00	0.37 to 24.43	0.306	2.54	0.30 to 21.36	0.390
Class III	12.67	1.18 to 136.31	0.036	12.26	1.08 to 138.65	0.043
Primary tumor status (uncontrolled vs. controlled)	1.46	0.37 to 5.80	0.588			
Extracranial metastases (present vs. absent)	0.71	0.23 to 2.19	0.549			
Number of BM (>3 vs. ≦3)	1.02	0.33 to 3.14	0.970			
Size of largest BM (mm)	1.01	0.97 to 1.05	0.560			
Hemorrhagic BM (yes vs. no)	0.76	0.23 to 2.58	0.664			
Total dose (>40 Gy_2_ vs. ≦40 Gy_2_)	0.86	0.23 to 3.28	0.825			
EGFR TKI during RT (yes vs. no)	1.32	0.44 to 3.97	0.620			
Type of EGFR TKI^§^ (erlotinib vs. gefitinib)	1.39	0.16 to 11.98	0.764			
Chemotherapy during RT (yes vs. no)	0.95	0.26 to 3.46	0.940			

Abbreviations: *HR* hazard ratio, *CI* confidence interval, *ECOG* Eastern Cooperative Oncology Group, *RPA* recursive partitioning analysis, *BM* brain metastases, *Gy*_*2*_ biologically equivalent dose equal to fraction size of 2 Gy, *TKI* tyrosine kinase inhibitor.

† *n* = 30.

§ *n* = 19.

‡ *P* for linear trend.

## Discussion

Our study intended to address the relationship between EGFR mutations and RT response in NSCLC, and concluded that NSCLC with mutant EGFR is more sensitive to brain RT in patients with brain metastases. Although EGFR overexpression is associated with radioresistance in cancer [13-16], EGFR mutations in NSCLC have been shown to confer radiosensitivity *in vitro*[17]. NSCLC cell lines with exon 19 deletions or L858R point mutation exhibit characteristics of radiosensitive phenotype, such as delayed double-strand DNA break repair and increased radiation-induced apoptosis. The radiosensitivity is independent of mutations in p53 or at EGFR residue 790 (T790M). Upon irradiation, the clonogenic survival for NSCLCs with EGFR mutations is reduced by up to 500- to 1000-fold, as compared with those with the wild-type. Clinically, previous research on the relationship between EGFR mutations in NSCLC and the response to brain RT has discrepant findings. One study reported that NSCLC patients with mutant EGFR have a significantly higher response rate to whole brain RT than those with wild-type EGFR (54% vs. 24%; *p* = 0.045), judged by the interval change of neurological symptoms, performance status, and the use of corticosteroids [19]. Nevertheless, another study showed no significant difference in brain RT response among NSCLC patients with and without EGFR mutations (67% vs. 50%; *p* = 0.23) by assessing at least 1 post-treatment brain MRI scan [18]. By using regular intracranial imaging follow-ups, our study demonstrated that NSCLC patients with EGFR mutations have a higher radiographic response rate. The image-based intracranial response rate for patients with mutant EGFR was approximately 2-fold higher than that for the wild-type group (80% vs. 46%; *p* = 0.037).

In this study, we also found that NSCLC with mutant EGFR is associated with prolonged intracranial RPFS (21 vs. 12 months; *p* = 0.009) in patients with brain metastases, as compared with wild-type EGFR. This result echoes previous finding that the median time to intracranial progression is longer in NSCLC patients with EGFR mutations than in those without (12.4 vs. 8.4 months; *p* = 0.39), judged by incidental findings of brain MRI [18]. Our study not only demonstrates the same trend by consecutive follow-up images but also suggests that the superior radiosensitivity in patients with mutant EGFR might contribute to a longer intracranial RPFS. In NSCLC patients with wild-type EGFR, more aggressive intracranial treatment for brain metastases, such as stereotactic radiosurgery or neurosurgical excision, should be considered, owing to the inferior treatment outcome of conventional brain RT. Furthermore, our study showed that a lower pre-treatment RPA class is associated with a longer intracranial RPFS (*p* = 0.026), but does not affect OS (*p* = 0.295). This finding is different from prior studies [6-8,12], in which a lower pre-treatment RPA class was associated with superior OS. This discrepancy might be explained by selection bias in our cohort. The majority of our patients (40 of 43) presented with adenocarcinoma and 70% had EGFR mutations. The median OS in our study was 15 months, which was longer than the 2 to 6 months reported in several previous studies [6-8,26-28]. This result echoes the findings of prior researches showing that adenocarcinoma [29] and EGFR mutations [18] are prognostic factors associated with long-term survival in lung cancer patients with brain metastases. In this patient subset, we showed that EGFR mutation status (*p* = 0.061) and pre-treatment performance status (*p* = 0.076) had a trend to predict OS. With recent advances in the management of NSCLC as well as the popularity of biomarker assessment, we suggest routine assessment of EGFR mutation status to provide personalized therapy and predict treatment outcome.

On the other hand, our study did not show a superior response to brain RT in NSCLC patients treated concurrently with an EGFR TKI (*p* = 0.864), despite the synergistic effect shown by previous studies [19,30]. In experimental models, the use of an EGFR TKI in combination with RT has been shown to increase antitumor activity by amplifying radiation-induced apoptosis and inhibiting tumor angiogenesis [30]. In one retrospective study, patients with concurrent EGFR TKI use were found to have a superior response rate to brain RT. [19]. However, pharmacokinetic analyses of EGFR TKIs report that only a tiny percentage (as low as 1%) of EGFR TKIs can penetrate into the cerebrospinal fluid, even in patients with brain metastases [31,32]. Therefore, an adequate EGFR TKI concentration for growth inhibition of mutant EGFR NSCLC cells might not be achieved in the brain by standard-dose EGFR TKI administration [33]. Thus, the clinical impact of combining an EGFR TKI with brain RT remains uncertain. In addition, variation in EGFR mutations might affect individual responses to EGFR TKI [34]. Although our retrospective study did not demonstrate a synergistic effect, it highlighted that future prospective trials are needed to elucidate the combined effect of EGFR TKIs and brain RT.

Due to the stringent inclusion criteria and retrospective nature of this study, the patient number was relatively small and most of the patients received a variety of treatments. Despite this weakness, there was a substantial difference between the survival curves of patients with and without EGFR mutations. In addition, possible discordance in EGFR expression between metastatic and primary tumors should be considered, since the majority of our samples used for EGFR mutation testing were from primary tissues. In NSCLC, disparities in EGFR mutations between metastatic and primary tumors were shown to have an 8.75% to 28% discordance rate [35-38]. However, it is not feasible to obtain brain tumor samples for genotyping in every patient with brain metastases. Recent advances in molecular imaging technologies such as positron emission tomography might be helpful for *in vivo* detection of EGFR distribution or activation in future studies [39,40]. Further prospective studies are imperative to clarify the degree of mutant EGFR that affects treatment outcome in NSCLC patients with brain metastases.

## Conclusions

In summary, our results suggest that mutant EGFR in NSCLC patients is an independent prognostic factor for better treatment response and longer intracranial RPFS following RT for brain metastases. Therefore, before brain RT, identification of EGFR mutation status is helpful in predicting treatment outcome.

## Competing interests

The authors declare no competing interests.

## Authors’ contributions

HLL, TSC and HEL provided the concept of the study and were involved in the study design, data analysis, writing and revision of the manuscript. HLL, TSC and LLT carried out all image evaluation and interpretation of the study. TSC, LLT, JTT, SWC, JFC and HWCL participated in clinical care and follow-up of the patients. All authors reviewed and approved the final manuscript.

## Supplementary Material

Additional file 1 **Table S1.** Radiographic response after brain RT based on RECIST criteria and associated EGFR mutation status. Click here for file

Additional file 2 **Table S2.** Univariate analysis of clinical characteristics in predicting overall survival. Click here for file

## References

[B1] Merchut MP Brain metastases from undiagnosed systemic neoplasms Arch Intern Med 1989 149 1076 1080 10.1001/archinte.1989.00390050066013 2719501

[B2] Parkin DM Bray F Ferlay J Global cancer statistics, 2002 CA Cancer J Clin 2005 55 74 108 10.3322/canjclin.55.2.74 15761078

[B3] Young K Palliation of brain and spinal cord metastases Perez and Brady's Principles and Practice of Radiation Oncology. 5th edition 2008 Philadelphia, USA: Lippincott Williams & Wilkins 1974 1985

[B4] Andrews DW Scott CB Sperduto PW Whole brain radiation therapy with or without stereotactic radiosurgery boost for patients with one to three brain metastases: phase III results of the RTOG 9508 randomised trial Lancet 2004 363 1665 1672 10.1016/S0140-6736(04)16250-8 15158627

[B5] Gaspar L Scott C Rotman M Recursive partitioning analysis (RPA) of prognostic factors in three Radiation Therapy Oncology Group (RTOG) brain metastases trials Int J Radiat Oncol Biol Phys 1997 37 745 751 10.1016/S0360-3016(96)00619-0 9128946

[B6] Gaspar LE Scott C Murray K Validation of the RTOG recursive partitioning analysis (RPA) classification for brain metastases Int J Radiat Oncol Biol Phys 2000 47 1001 1006 10.1016/S0360-3016(00)00547-2 10863071

[B7] Kepka L Cieslak E Bujko K Results of the whole-brain radiotherapy for patients with brain metastases from lung cancer: the RTOG RPA intra-classes analysis Acta Oncol 2005 44 389 398 10.1080/02841860510029699 16120548

[B8] Mehta MP Tsao MN Whelan TJ The American Society for Therapeutic Radiology and Oncology (ASTRO) evidence-based review of the role of radiosurgery for brain metastases Int J Radiat Oncol Biol Phys 2005 63 37 46 10.1016/j.ijrobp.2005.05.023 16111570

[B9] Kocher M Soffietti R Abacioglu U Adjuvant whole-brain radiotherapy versus observation after radiosurgery or surgical resection of one to three cerebral metastases: Results of the EORTC 22952-26001 study J Clin Oncol 2011 29 134 141 10.1200/JCO.2010.30.1655 21041710 PMC3058272

[B10] Saito EY Viani GA Ferrigno R Whole brain radiation therapy in management of brain metastasis: results and prognostic factors Radiat Oncol 2006 1 20 10.1186/1748-717X-1-20 16808850 PMC1526744

[B11] Sperduto PW Chao ST Sneed PK Diagnosis-specific prognostic factors, indexes, and treatment outcomes for patients with newly diagnosed brain metastases: a multi-institutional analysis of 4,259 patients Int J Radiat Oncol Biol Phys 2010 77 655 661 10.1016/j.ijrobp.2009.08.025 19942357

[B12] Sperduto PW Kased N Roberge D Summary report on the graded prognostic assessment: An accurate and facile diagnosis-specific tool to estimate survival for patients with brain metastases J Clin Oncol 2012 30 419 425 10.1200/JCO.2011.38.0527 22203767 PMC3269967

[B13] Baumann M Molecular-targeted agents for enhancing tumour response Basic Clinical Radiobiology. 4th edition 2009 London, UK: Hodder Arnold 287 300

[B14] Giralt J de las Heras M Cerezo L The expression of epidermal growth factor receptor results in a worse prognosis for patients with rectal cancer treated with preoperative radiotherapy: a multicenter, retrospective analysis Radiother Oncol 2005 74 101 108 10.1016/j.radonc.2004.12.021 15816107

[B15] Liang K Ang KK Milas L The epidermal growth factor receptor mediates radioresistance Int J Radiat Oncol Biol Phys 2003 57 246 254 10.1016/S0360-3016(03)00511-X 12909240

[B16] Sheridan MT O'Dwyer T Seymour CB Potential indicators of radiosensitivity in squamous cell carcinoma of the head and neck Radiat Oncol Investig 1997 5 180 186 10.1002/(SICI)1520-6823(1997)5:4<180::AID-ROI3>3.0.CO;2-U 9327497

[B17] Das AK Sato M Story MD Non-small-cell lung cancers with kinase domain mutations in the epidermal growth factor receptor are sensitive to ionizing radiation Cancer Res 2006 66 9601 9608 10.1158/0008-5472.CAN-06-2627 17018617

[B18] Eichler AF Kahle KT Wang DL EGFR mutation status and survival after diagnosis of brain metastasis in nonsmall cell lung cancer Neuro Oncol 2010 12 1193 1199 10.1093/neuonc/noq076 20627894 PMC3098020

[B19] Gow CH Chien CR Chang YL Radiotherapy in lung adenocarcinoma with brain metastases: effects of activating epidermal growth factor receptor mutations on clinical response Clin Cancer Res 2008 14 162 168 10.1158/1078-0432.CCR-07-1468 18172267

[B20] Lee CN Yu MC Bai KJ NAT2 fast acetylator genotypes are associated with an increased risk for lung cancer with wildtype epidermal growth factor receptors in Taiwan Lung Cancer 2009 64 9 12 10.1016/j.lungcan.2008.07.001 18706736

[B21] Eisenhauer EA Therasse P Bogaerts J New response evaluation criteria in solid tumours: revised RECIST guideline (version 1.1) Eur J Cancer 2009 45 228 247 10.1016/j.ejca.2008.10.026 19097774

[B22] Trotti A Colevas AD Setser A CTCAE v3.0: development of a comprehensive grading system for the adverse effects of cancer treatment Semin Radiat Oncol 2003 13 176 181 10.1016/S1053-4296(03)00031-6 12903007

[B23] Kosaka T Yatabe Y Endoh H Mutations of the epidermal growth factor receptor gene in lung cancer: biological and clinical implications Cancer Res 2004 64 8919 8923 10.1158/0008-5472.CAN-04-2818 15604253

[B24] Sonobe M Manabe T Wada H Mutations in the epidermal growth factor receptor gene are linked to smoking-independent, lung adenocarcinoma Br J Cancer 2005 93 355 363 10.1038/sj.bjc.6602707 16052218 PMC2361570

[B25] Tam IY Chung LP Suen WS Distinct epidermal growth factor receptor and KRAS mutation patterns in non-small cell lung cancer patients with different tobacco exposure and clinicopathologic features Clin Cancer Res 2006 12 1647 1653 10.1158/1078-0432.CCR-05-1981 16533793

[B26] Bergqvist M Brattström D Bennmarker H Irradiation of brain metastases from lung cancer: a retrospective study Lung Cancer 1998 20 57 63 10.1016/S0169-5002(98)00015-4 9699188

[B27] Lagerwaard FJ Levendag PC Nowak PJ Identification of prognostic factors in patients with brain metastases: a review of 1292 patients Int J Radiat Oncol Biol Phys 1999 43 795 803 10.1016/S0360-3016(98)00442-8 10098435

[B28] Rodrigus P de Brouwer P Raaymakers E Brain metastases and non-small cell lung cancer. prognostic factors and correlation with survival after irradiation Lung Cancer 2001 32 129 136 10.1016/S0169-5002(00)00227-0 11325483

[B29] Noterman J Hildebrand J Rocmans P [Long-term survival after surgery of solitary cerebral metastasis of lung cancer. Clinical case and review of the literature] Neurochirurgie 1990 36 308 311 2267045

[B30] Huang SM Li J Armstrong EA Modulation of radiation response and tumor-induced angiogenesis after epidermal growth factor receptor inhibition by ZD1839 (Iressa) Cancer Res 2002 62 4300 4306 12154033

[B31] Jackman DM Holmes AJ Lindeman N Response and resistance in a non-small-cell lung cancer patient with an epidermal growth factor receptor mutation and leptomeningeal metastases treated with high-dose gefitinib J Clin Oncol 2006 24 4517 4520 10.1200/JCO.2006.06.6126 16983123

[B32] Lassman AB Rossi MR Raizer JJ Molecular study of malignant gliomas treated with epidermal growth factor receptor inhibitors: tissue analysis from North American Brain Tumor Consortium Trials 01-03 and 00-01 Clin Cancer Res 2005 11 7841 7850 10.1158/1078-0432.CCR-05-0421 16278407

[B33] Clarke JL Pao W Wu N High dose weekly erlotinib achieves therapeutic concentrations in CSF and is effective in leptomeningeal metastases from epidermal growth factor receptor mutant lung cancer J Neurooncol 2010 99 283 286 10.1007/s11060-010-0128-6 20146086 PMC3973736

[B34] Yang CH Yu CJ Shih JY Specific EGFR mutations predict treatment outcome of stage IIIB/IV patients with chemotherapy-naive non-small-cell lung cancer receiving first-line gefitinib monotherapy J Clin Oncol 2008 26 2745 2753 10.1200/JCO.2007.15.6695 18509184

[B35] Gow CH Chang YL Hsu YC Comparison of epidermal growth factor receptor mutations between primary and corresponding metastatic tumors in tyrosine kinase inhibitor-naive non-small-cell lung cancer Ann Oncol 2009 20 696 702 10.1093/annonc/mdn679 19088172

[B36] Kalikaki A Koutsopoulos A Trypaki M Comparison of EGFR and K-RAS gene status between primary tumours and corresponding metastases in NSCLC Br J Cancer 2008 99 923 929 10.1038/sj.bjc.6604629 19238633 PMC2538768

[B37] Park S Holmes-Tisch AJ Cho EY Discordance of molecular biomarkers associated with epidermal growth factor receptor pathway between primary tumors and lymph node metastasis in non-small cell lung cancer J Thorac Oncol 2009 4 809 815 10.1097/JTO.0b013e3181a94af4 19487967

[B38] Sun L Zhang Q Luan H Comparison of KRAS and EGFR gene status between primary non-small cell lung cancer and local lymph node metastases: implications for clinical practice J Exp Clin Cancer Res 2011 30 30 10.1186/1756-9966-30-30 21414214 PMC3069944

[B39] Mishani E Hagooly A Strategies for molecular imaging of epidermal growth factor receptor tyrosine kinase in cancer J Nucl Med 2009 50 1199 1202 10.2967/jnumed.109.062117 19617320

[B40] Pantaleo MA Nannini M Maleddu A Experimental results and related clinical implications of PET detection of epidermal growth factor receptor (EGFr) in cancer Ann Oncol 2009 20 213 226 18842614 10.1093/annonc/mdn625

