# Supplementary material for: EGFR mutations are associated with favorable intracranial response and progression-free survival following brain irradiation in non-small cell lung cancer patients with brain metastases
Source: Radiat Oncol. 2012 Oct 30;7:181. doi: 10.1186/1748-717X-7-181 (PMC3549835; doi:10.1186/1748-717X-7-181)
Supplement: Additional file 1 — Table S1. Radiographic response after brain RT based on RECIST criteria and associated EGFR mutation status. [file 1748-717X-7-181-S1.doc]

**Supplementary Table 1**

Radiographic response after brain RT based on RECIST criteria and associated EGFR mutation status.

|  | **Total**  **(*N* = 43)** | **EGFR mutation status** | |  |
| --- | --- | --- | --- | --- |
| **Brain RT response** |  | **Positive**  **(*****n* = 30)** | **Negative**  **(*n* = 13)** | ***P*** |
| No | 13 (30) | 6 (20) | 7 (54) | 0.037 |
| PD | 2 | 0 | 2 |  |
| SD | 11 | 6 | 5 |  |
| Yes | 30 (70) | 24 (80) | 6 (46) |  |
| PR | 25 | 21 | 4 |  |
| CR | 5 | 3 | 2 |  |

Abbreviations: PD, progressive disease; SD, stable disease; PR, partial response; CR, complete response.

Categorical data were presented as number (percentage).
