# Supplementary material for: EGFR mutations are associated with favorable intracranial response and progression-free survival following brain irradiation in non-small cell lung cancer patients with brain metastases
Source: Radiat Oncol. 2012 Oct 30;7:181. doi: 10.1186/1748-717X-7-181 (PMC3549835; doi:10.1186/1748-717X-7-181)
Supplement: Additional file 2 — Table S2. Univariate analysis of clinical characteristics in predicting overall survival. [file 1748-717X-7-181-S2.doc]

**Supplementary Table 2**

Univariate analysis of clinical characteristics in predicting overall survival.

|  | **Univariate analysis** | | |
| --- | --- | --- | --- |
| **Characteristics** | **HR** | **95% CI of HR** | ***P*** |
| Age, years (≧60 vs. ＜60) | 1.56 | 0.75 to 3.25 | 0.235 |
| Gender (male vs. female) | 1.04 | 0.51 to 2.11 | 0.915 |
| Smoking history  (ever vs. never) | 1.44 | 0.67 to 3.07 | 0.349 |
| EGFR mutation status (positive vs. negative) | 0.47 | 0.22 to 1.03 | 0.061 |
| Mutant EGFR^†^  (exon 21 vs. exon 19) | 0.88 | 0.37 to 2.11 | 0.780 |
| ECOG performance status | 1.39 | 0.97 to 2.01 | 0.076 |
| RPA class |  |  | 0.295^‡^ |
| Class I (reference) | 1 | -- | -- |
| Class II | 1.83 | 0.54 to 6.18 | 0.332 |
| Class III | 2.14 | 0.53 to 8.61 | 0.286 |
| Primary tumor status (uncontrolled vs. controlled) | 1.82 | 0.88 to 3.77 | 0.105 |
| Extracranial metastases (present vs. absent) | 1.15 | 0.53 to 2.49 | 0.724 |
| Number of BM (＞3 vs. ≦3) | 1.08 | 0.52 to 2.23 | 0.840 |
| Size of largest BM (mm) | 0.99 | 0.96 to 1.02 | 0.987 |
| Hemorrhagic BM (yes vs. no) | 0.55 | 0.24 to 1.25 | 0.152 |
| Total dose (＞40 Gy_2_ vs. ≦40 Gy_2_) | 1.43 | 0.69 to 2.96 | 0.337 |
| EGFR TKI during RT (yes vs. no) | 0.85 | 0.40 to 1.78 | 0.664 |
| Type of EGFR TKI^§^ (erlotinib vs. gefitinib) | 0.66 | 0.23 to 1.96 | 0.458 |
| Chemotherapy during RT (yes vs. no) | 1.28 | 0.52 to 3.18 | 0.591 |
| Brain RT response (yes vs. no) | 0.59 | 0.28 to 1.23 | 0.159 |

Abbreviations: HR, hazard ratio; CI, confidence interval; ECOG, Eastern Cooperative Oncology Group; RPA, recursive partitioning analysis; BM, brain metastases; Gy_2_, biologically equivalent dose equal to fraction size of 2 Gy; TKI, tyrosine kinase inhibitor.

† *n* = 30.

§ *n* = 19.

‡ *P* for linear trend.
